# Development and Application of Nano-Micro Sealant for Water-Based Drilling Fluids in Deep Shale Gas Formations of the Sichuan-Chongqing Region

**DOI:** 10.3390/gels12060475

**Published:** 2026-05-29

**Authors:** Jiali Wang, Long Chen, Jiayin Zhang, Yu Sang, Yunhai Zhao, Hui Mao

**Affiliations:** 1 Oil & Gas Technology Research Institute, Petrochina Southwest Oil & Gasfield Company, Chengdu 610017, China; wangjiali2022@petrochina.com.cn (J.W.); chenlong01@petrochina.com.cn (L.C.); zhangjiayin@petrochina.com.cn (J.Z.); sangy@petrochina.com.cn (Y.S.); zyh11230715@163.com (Y.Z.); 2 College of Energy (College of Modern Shale Gas Industry), Chengdu University of Technology, Chengdu 610059, China; 3 State Key Laboratory of Oil and Gas Reservoir Geology and Exploitation, Chengdu University of Technology, Chengdu 610059, China

**Keywords:** colloidal, hydrogels, core–shell structure, water-based drilling fluid, nanomicron plug, shale gas

## Abstract

To address wellbore instability and the technical challenges associated with high-density water-based drilling fluid loss control in deep shale gas formations of the Sichuan-Chongqing region in China, a novel nano-micro sealant designated CLG-Seal was synthesized via molecular structural optimization. The molecular structure of newly developed CLG-Seal exhibits distinct core–shell structural characteristics. The inorganic nano-silica constitutes the rigid core of CLG-Seal, which guarantees its plugging performance. The hydrophobically associating polymer which is coated on the surface of nano-silica constructs the flexible shell of CLG-Seal, endowing the CLG-Seal with excellent gel-forming capacity, adhesion film-forming capacity, deformability and perfect dispersibility. Transmission electron microscopy and scanning electron microscopy were employed to characterize the morphology of the CLG-Seal nanomicron-scale plugging agent. The sealing performance and underlying mechanisms of CLG-Seal were subsequently evaluated via particle plugging apparatus tests, displacement experiments, and etched glass micromodel simulations. Field trials conducted in the third section of Well WY3-2-3HF validated the application effectiveness of this agent in drilling fluid systems. The results indicate that the nano-micro sealant CLG-Seal exhibits a median particle size of D_50_ is 146 nm, which can be modulated by adjusting the synthesis conditions. The nano-micro sealant CLG-Seal significantly mitigates fluid loss in low-permeability microfractures and fissures. Notably, a concentration of merely 3% is sufficient to achieve optimal nano-micro plugging performance. The results of the mechanism study indicate that while the CLG-Seal particles are close to each other, the polymer chains with flexible long chain structure which are coated on the surface of nano-silica constructs tend to be intertwined, forming a cross-linked network structure of gel film, thereby increasing the interaction between nano-micron particles and forming an impermeable plugging film. In addition, due to the nanoscale effect, the CLG-Seal has a strong tendency to adsorb onto the surface of shale rock through hydrogen bonding with the shale matrix. The hydrophobically associating polymer with high elastic modulus and excellent mechanical properties can enhance the pressure-bearing capacity of the filter cake through elastic deformation. Therefore, these nano-micron particles can form a strong sealing film on the filter cake and at the micropores of shale rock, thereby creating a dense mud cake on the outside of the shale formation. Field trial results demonstrate that the incorporation of the nano-micro sealant CLG-Seal into the drilling fluid for the third section of Well WY3-2-3HF reduced the PPA fluid loss to 4.6 mL. This value represents a substantial reduction compared to adjacent wells and signifies a remarkable improvement over the drilling fluids previously employed in the Longmaxi Formation of this block. Furthermore, the treated drilling fluid exhibited a superior filtration control pressure capacity of 10.5 MPa. The operation was completed successfully without any lost circulation or wellbore instability, and achieved a drilling footage of 42 h with an average penetration rate of 7.81 m/h. The mud weight was reduced by approximately 0.08–0.10 g/cm^3^ compared to offset wells. These results confirm the excellent application efficiency of the newly developed CLG-Seal in field operations.

## 1. Introduction

The Sichuan-Chongqing region is not only a pioneer in China’s shale gas development but also hosts the Changning-Weiyuan National Shale Gas Demonstration Zone. These factors underscore the region’s critical strategic importance in the nation’s shale gas exploitation landscape. However, the deep shale formations in this region are characterized by well-developed fractures and pronounced water sensitivity. During drilling operations, these geological features frequently induce wellbore instability incidents, such as borehole collapse and lost circulation, leading to severe operational complications, including excessive fluid loss, pipe sticking, and drill string burying. Furthermore, challenges related to high frictional resistance, cuttings transport, and reservoir damage are particularly prominent in long horizontal sections [1,2,3,4]. Consequently, the performance of the drilling fluid system plays a pivotal role in determining drilling efficiency, mitigating non-productive time (NPT), and ensuring effective reservoir protection. Consequently, oil-based drilling fluids (OBM) were historically the preferred choice for shale gas horizontal drilling due to their inherent superiority in inhibiting shale hydration and swelling, coupled with excellent lubricity. The OBM system offers straightforward formulation and manageable rheology, facilitating effective hole cleaning and reduced annular pressure loss. However, OBM suffers from significant drawbacks, including high costs, poor environmental compatibility, and challenges in managing oil-contaminated cuttings. Crucially, in formations characterized by micro-fractures and micropores, OBM is often more prone to fluid loss than water-based mud (WBM), leading to severe economic losses. Therefore, developing cost-effective water-based drilling fluid technologies capable of substituting OBM in shale gas reservoirs is imperative [5,6,7].

Field operations demonstrate that due to the pronounced water sensitivity and well-developed micro-fractures/micropores of shale reservoirs, wellbore instability incidents—such as sloughing and borehole collapse—frequently occur during water-based drilling fluid (WBM) operations, often resulting in complex downhole situations like pipe sticking. Simultaneously, the severe hydration and dispersion of drill cuttings from shale formations into the WBM lead to significant technical challenges, including the loss of rheological and filtration control and deteriorated lubricity [8].

In view of the characteristics of microscopic pore structures in shale gas reservoirs, researchers have initiated investigations into incorporating nanoparticles into drilling fluids to enhance the sealing efficiency of nano-scale pores and microfractures, yielding preliminary success. Among these, inorganic nanoparticles—such as graphite, silica, calcium carbonate, and iron oxides—have been among the earliest candidates explored for developing water-based drilling fluid additives, owing to their low cost and facile synthesis [9,10,11]. Previous studies report that nano-silica can effectively infiltrate shale micro-pores and throats, forming compact internal and external filter cakes that significantly reduce shale permeability. Specifically, nano-silica has been shown to reduce water invasion in the Atoka shale by up to 98%. Scanning electron microscopy (SEM) observations reveal that these nanoparticles penetrate and block pore throats to form a permanent filter cake. Consequently, the sealing efficiency is highly dependent on the nanoparticle concentration and the matching degree between particle size distribution and the diameter of shale pore throats. Based on the anti-agglomeration mechanism, Qiu et al. analyzed the dispersion effects of mechanical shearing and dispersants on nano-calcium carbonate, identifying the reduction in surface tension and steric hindrance as the primary mechanisms for stabilizing the dispersion system; consequently, they prepared a composite dispersant and optimized the relevant dispersion conditions. Subsequent studies have further expanded on nanoparticle applications [10]. Paiaman et al. incorporated nano-silica into water-based drilling fluids (WBM), demonstrating a marked reduction in shale permeability alongside significant enhancements in rheological and lubricating properties, while Wu Yuanpeng et al. fabricated a poly(2-acrylamide-2-methylpropanesulfonic acid)/SiO_2_ composite nanoparticle capable of effectively sealing nano-scale pores within filter cakes while exhibiting excellent thermal stability [12]. Similarly, Hoelscher et al. modified WBM with surface-functionalized nano-SiO_2_ to improve fluid loss control, and Javeri et al. utilized silica nanoparticles to enhance filter cake quality and mitigate the risk of differential pipe sticking. Extending this research to magnetic nanomaterials, Yue and Wu synthesized Fe_3_O_4_ nanoparticles (average size: 9.5 nm) via thermal decomposition, which demonstrated superior plugging performance along with favorable magnetic responsiveness and salt tolerance [13].

Organic nanoparticles, characterized by their compressibility and deformability under specific temperature and pressure conditions, can penetrate the formation’s primary sealing zone to reinforce the plugging of micro-pores. This capability enhances the compactness and pressure-bearing capacity of the filter cake, rendering it a promising candidate for improving the plugging performance of drilling fluids. Bai Xiaodong et al. employed emulsion polymerization to synthesize polymethyl methacrylate (PMMA) nanoparticles, which significantly enhanced the inhibitory properties and fluid loss control of drilling fluids. Concurrently, Wang Weiji et al. prepared nano-polymer microsphere sealants via the same technique using styrene and methyl methacrylate (MMA) monomers with potassium persulfate as an initiator; these microspheres effectively retarded shale pressure transmission and fluid invasion, reducing shale permeability by over 95%, thereby offering a promising solution for shale wellbore stability [14]. Extending this approach, Xu et al. fabricated nano-polymer microspheres using styrene, butyl acrylate, and acrylic acid with a composite emulsifier, forming a compact sealing film on the shale surface that effectively blocked the formation and significantly slowed pressure transmission rates [15]. Furthermore, Qiu developed a nano-emulsion that markedly improved reservoir protection performance while simultaneously exhibiting excellent lubricity and plugging efficiency [16].

Furthermore, researchers have actively explored organic/inorganic nanocomposites to enhance drilling fluid performance. Ke Yangchuan prepared a core–shell nanocomposite using montmorillonite and polyacrylamide, which significantly improved rheological properties and fluid loss control [17]. Similarly, Jiang fabricated a nano-SiO_2_/polymer composite fluid loss additive via fine emulsion polymerization; this additive exhibited excellent high-temperature filtration control, film-forming water-blocking capabilities, and robust resistance to salt and calcium contamination [18]. In a related study, Shen and Jiang developed a nano-bentonite composite via the intercalation of clay with acrylamide and its derivatives, achieving superior thermal stability, contamination resistance, and cuttings transport capacity. Additionally, Zong engineered a polymer/clay ultrafine particle composite that markedly enhanced fluid loss control and lubricity. Extending this to advanced polymerization techniques, Qu synthesized a poly(styrene-b-acrylamide)/montmorillonite nanocomposite via reversible addition-fragmentation chain transfer (RAFT) radical polymerization, endowing the drilling fluid with exceptional high-temperature stability and superior filtration performance [19].

Nano-micro sealants constitute a class of critical additives in water-based drilling fluids (WBM) for deep shale reservoirs; characterized by strong hydrophilicity and an expanded hydrodynamic volume upon hydration, they effectively enhance the plugging performance of the fluid while simultaneously controlling fluid loss. Given that hydrophobic association polymers offer excellent thermal stability, salt tolerance, and contamination resistance, making them promising candidates for deep and ultra-deep well drilling [20], and considering that nano-silica provides high surface energy, rigidity, and dimensional/thermal stability, a composite architecture integrating these materials is highly desirable. By fabricating a nano-micro sealant featuring a nano-silica core and a polymeric shell, it is possible to synergistically combine the rigidity and stability of the inorganic phase with the toughness, processability, and interfacial properties of the polymer matrix [11]. Addressing the finer-scale nano-micro fractures prevalent in the deep shale formations of the Sichuan-Chongqing region, this study endeavors to develop a novel polymer-based nano-micro sealant. This work aims to upgrade the plugging performance of current field WBM systems, thereby providing an effective solution to wellbore instability challenges in deep shale gas exploration.

## 2. Result and Discussion

### 2.1. Transmission Electron Microscopy (TEM) Analysis

**Figure 1:**
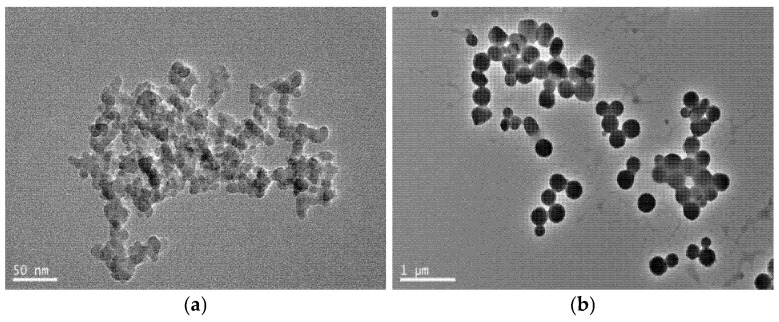
TEM characterization of (**a**) nano-silica and (**b**) the newly developed CLG-Seal in aqueous solution.

Transmission electron microscopy (TEM) was employed to characterize the dispersion morphology of nano-silica and CLG-Seal in aqueous solution. Figure 1a displays the TEM image of the nano-silica aqueous solution, while Figure 1b presents the TEM image of the newly developed CLG-Seal in solution.

As illustrated in Figure 1a, hydrophilic nano-silica exhibits significant agglomeration in aqueous solution. In contrast, Figure 1b reveals that the CLG-Seal comprises granular nano-silica particles with diameters ranging from 150 nm to 250 nm, demonstrating excellent dispersion in the aqueous system.

### 2.2. Scanning Electron Microscopy (SEM) Analysis

**Figure 2:**
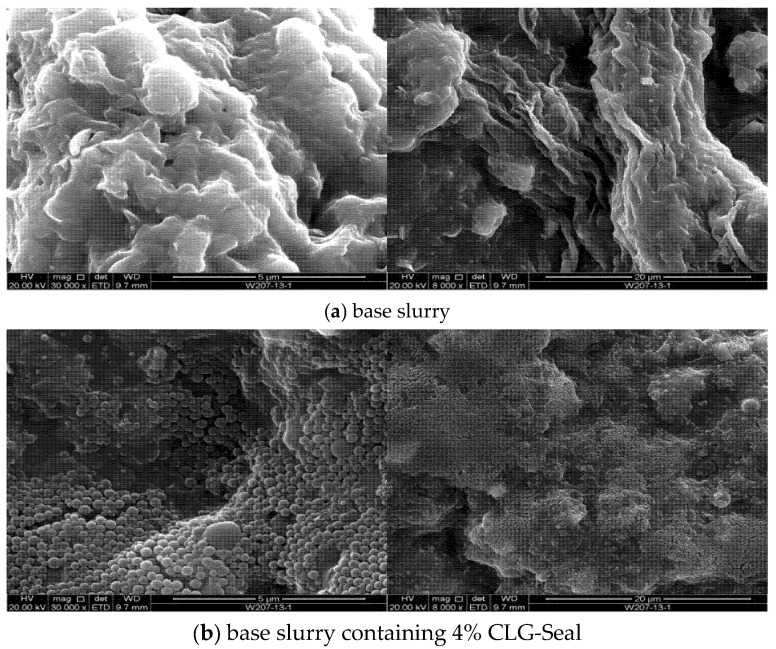
SEM characterization of filter cake surfaces: (**a**) base slurry; (**b**) base slurry containing 4% CLG-Seal.

Field-emission scanning electron microscopy (FE-SEM) was employed to characterize the surface morphology of API filter cakes prepared from a base fluid containing 4% CLG-Seal. Figure 2 presents the FE-SEM image of the synthesized CLG-Seal distributed on the filter cake surface.

Figure 2 reveals that the filter cake formed by the 4% base slurry exhibits visible micro-fractures at the macro scale, with inter-particle gaps between clay platelets measuring approximately 2–3 μm under higher magnification. In contrast, the incorporation of the newly developed CLG-Seal into the base slurry demonstrates that nano-micro particles actively participate in the filter cake formation. These particles arrange themselves in a tightly packed configuration on the cake surface, effectively minimizing porosity. These observations collectively indicate that the synthesized CLG-Seal possesses exceptional nano-micro plugging performance.

### 2.3. Particle Size Distribution (PSD) Analysis

**Figure 3:**
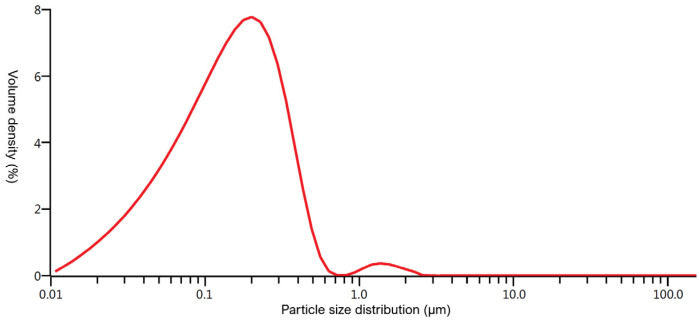
The PSD analysis results of a 0.1 wt% CLG-Seal aqueous solution.

The particle size distribution (PSD) of a 0.1 wt% CLG-Seal aqueous solution was measured, and the results are presented in Figure 3.

The experimental results indicate that CLG-Seal exhibits a continuous particle size distribution, with a median particle size (D_50_) of 146 nm, D_10_ of 40 nm and D_90_ of 344 nm. Furthermore, it was observed that the D_50_ of the newly developed nano-micro sealant is tunable.

### 2.4. Evaluation of PPA Sealing Performance

**Figure 4:**
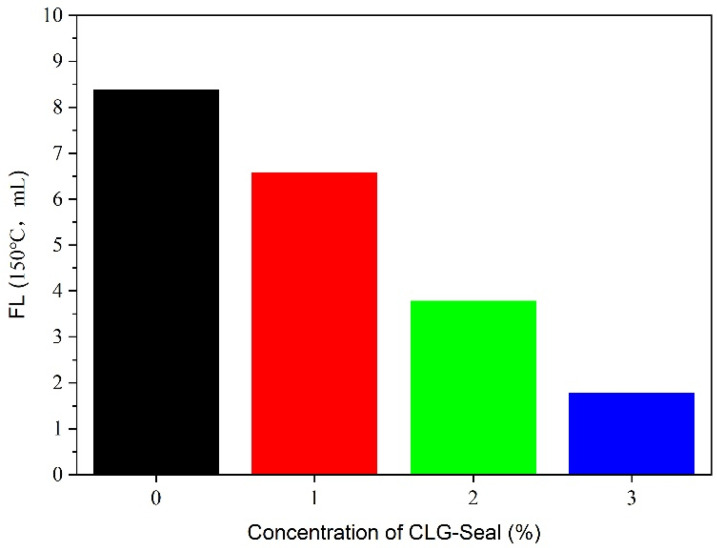
PPA test results of experimental fluids after hot rolling.

**Figure 5:**
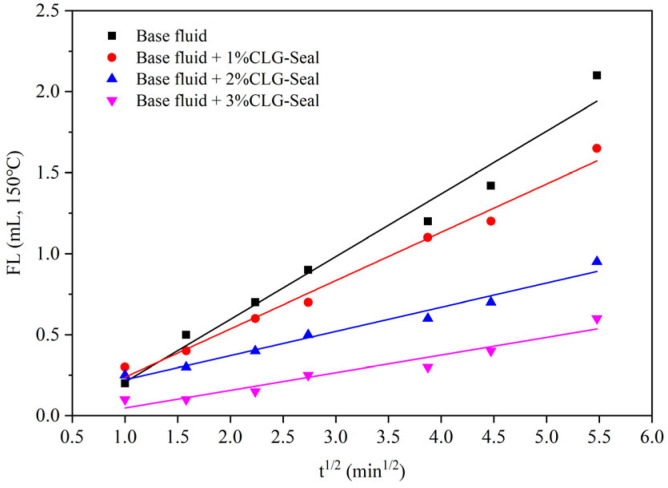
Filtrate volume versus the square root of time for experimental fluids after hot rolling.

The experimental results are presented in Figure 4 and Figure 5. As illustrated in Figure 4, the fluid loss in low-permeability sand disks, after hot rolling at 150 °C, exhibits a consistent downward trend with increasing concentrations of the newly developed CLG-Seal. Specifically, the fluid loss was 8.4 mL for the base fluid without the additive, whereas it was reduced to merely 1.8 mL with the addition of 3% CLG-Seal. These findings demonstrate that the nano-micro sealant significantly mitigates fluid loss in low-permeability microfractures and fissures, indicating that robust plugging performance can be achieved at a dosage of merely 3%.

Figure 5 demonstrates a linear correlation between PPA sand disk fluid loss and the square root of time. The y-intercept of the fitted line represents the instantaneous fluid loss, while the slope corresponds to the static filtration rate; notably, the total PPA fluid loss equals twice the value at 30 min. Further analysis reveals that the newly developed CLG-Seal significantly reduces both instantaneous fluid loss and static filtration rate. This indicates that during drilling in naturally fractured formations, CLG-Seal rapidly forms a compact, pressure-bearing sealing layer within micro-fractures or pores (nano-micro pores), thereby enhancing the plugging performance of the drilling fluid and realizing the objective of active lost-circulation prevention.

**Figure 6:**
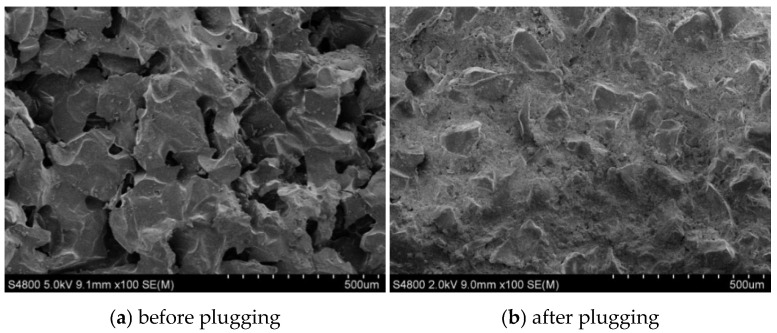
SEM images of the PPA sand disk after plugging.

Figure 6 displays SEM images of the cross-section of the sand disk (Filter No. 12114301d) before and after the permeability plugging test. As observed in Figure 6, CLG-Seal particles penetrate the micro-pores of the low-permeability sand disk, and a high density of these particles is evident within the sealing layer. This further confirms that the nano-micro sealant can promptly access micro-fractures and pores, thereby forming a compact, pressure-bearing plugging layer at the nano-micro scale.

### 2.5. Evaluation of Displacement Plugging Performance

**Figure 7:**
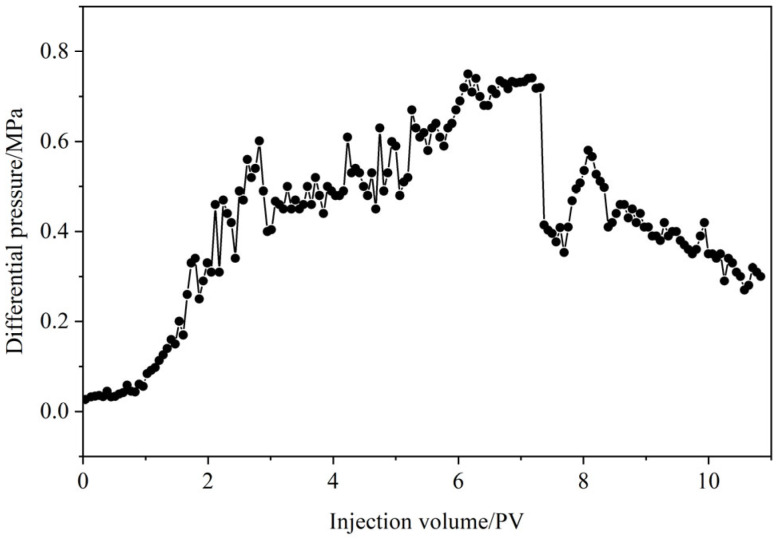
Displacement plugging performance of CLG-Seal in artificial cores.

**Figure 8:**
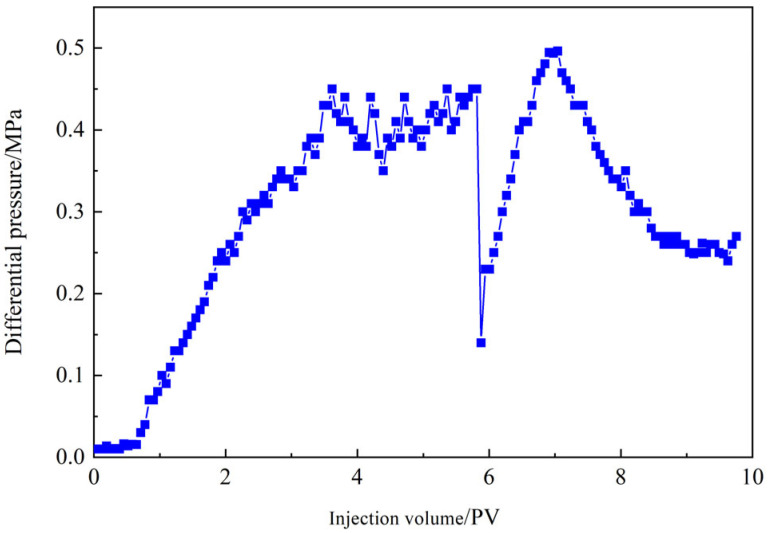
Displacement plugging performance of N-Seal in artificial cores.

**Figure 9:**
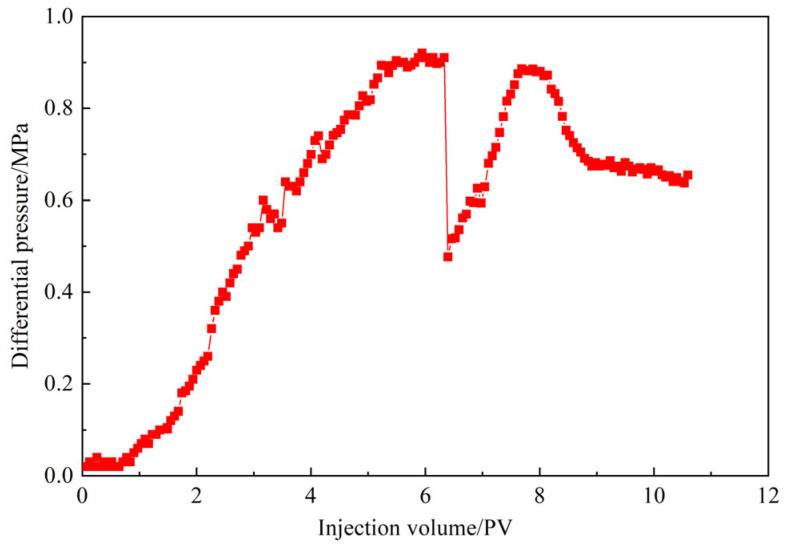
Displacement plugging performance of SD-Seal in artificial cores.

The displacement plugging performance of CLG-Seal was evaluated alongside two commercial nano-micro sealants, N-Seal and SD-Seal, with the results presented in Figure 7, Figure 8 and Figure 9. Table 1 summarizes the calculated resistance factors and residual resistance factors obtained from the displacement plugging experiments.

As indicated by Figure 7 and Table 1, the displacement pressure for the base fluid was 0.15 MPa, whereas it increased to 0.77 MPa following the addition of 4% CLG-Seal. The subsequent water flooding pressure was recorded at 0.46 MPa, yielding a resistance factor of 5.13 and a residual resistance factor of 3.07. These results demonstrate the superior plugging capability of the nano-micro particles within pores and fractures.

This performance is primarily attributed to the unique structure of the newly developed CLG-Seal, which features a deformable soft shell encapsulating a rigid nano-silica core. Benefiting from the core’s small particle size, high rigidity, excellent thermal stability, and resistance to crushing and hydration, the sealant can rapidly plug formation micro-pores and fractures matching its dimensions upon drilling into new formations. The particles aggregate into clusters and strings, adsorbing, bridging, and plugging at pore throats to form a thin yet dense isolation layer.

Furthermore, due to the high surface energy of these ultrafine particles, intermolecular interactions facilitate the bridging and cross-linking of particles with varying sizes, enabling the sealing of pores larger than the individual particles themselves. Consequently, this bridging and filling mechanism allows for the rapid formation of a robust and compact filter cake. Moreover, the extension of the non-polar segments within the polymer matrix creates a barrier that impedes the passage of polar water molecules. This results in a thin, dense filter cake that minimizes high-temperature, high-pressure (HTHP) fluid loss, enhances the pressure-bearing capacity of the formation, and ultimately contributes to reservoir protection and wellbore stability.

As shown in Figure 8 and Table 1, the introduction of 4% N-Seal into the base fluid resulted in a displacement pressure of 0.45 MPa and a subsequent water flooding pressure of 0.50 MPa, yielding a resistance factor of 3.00 and a residual resistance factor of 3.33. These results indicate that the commercial nano-micro sealant N-Seal imparts a moderate plugging capability to the freshwater base fluid; however, its performance remains inferior to that of the newly developed CLG-Seal under identical conditions.

As evidenced by Figure 9 and Table 1, the incorporation of 4% SD-Seal into the base fluid resulted in a displacement pressure of 0.31 MPa and a subsequent water flooding pressure of 0.29 MPa, yielding a resistance factor of 2.07 and a residual resistance factor of 1.93. These results suggest that while the domestic nano-micro sealant SD-Seal provides a certain degree of plugging capacity, its performance is still significantly inferior to that of the newly developed CLG-Seal.

In summary, the plugging performance of the newly developed CLG-Seal is superior to that of both N-Seal and SD-Seal. Its incorporation into the freshwater base fluid effectively facilitates the formation of a robust seal within micro-fractures and pores.

### 2.6. Evaluation of Plugging Performance in Etched Micromodel Fractures

**Figure 10:**
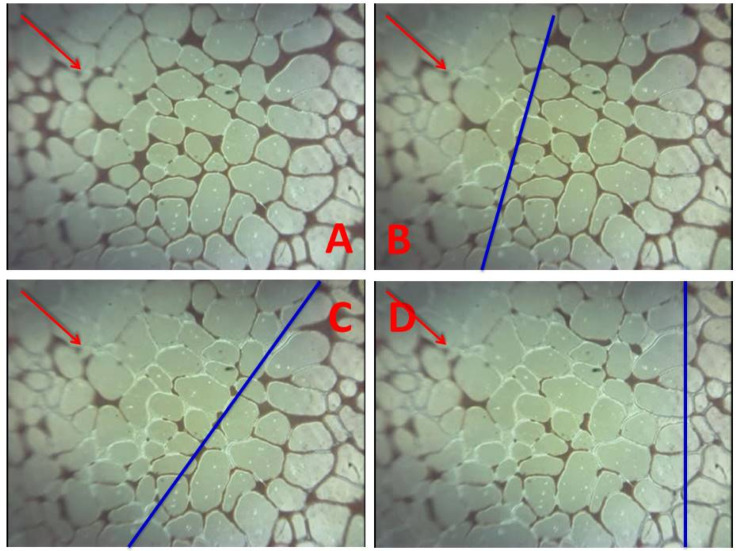
Microscopic plugging process of nano-micro pores by CLG-Seal.

The plugging performances in etched glass micromodels are shown in Figure 10, where the red arrows indicate the inlet and flow direction of the displacement fluid.

Figure 10A shows the etched glass micromodel saturated with deionized water under vacuum, revealing that water predominantly adheres to the micro-fractures and pores, thereby validating the model’s capability to simulate hydrophilic rock fractures. As illustrated in Figure 10B,C, the CLG-Seal dispersion was injected into the micromodel at a controlled backpressure of 1.5 MPa. Post-experiment analysis [Figure 10D] indicates that the area invaded by the fluid accounts for approximately 5/6 of the total model area, with no significant invasion observed in the remaining 1/6. These results demonstrate that the experimental fluid possesses a strong plugging capacity for micro-fractures. Furthermore, they confirm that the newly developed CLG-Seal effectively mitigates fluid loss in water-based drilling fluids by actively sealing formation micro-fractures.

### 2.7. Discussion on the Mechanism of CLG-Seal

**Figure 11:**
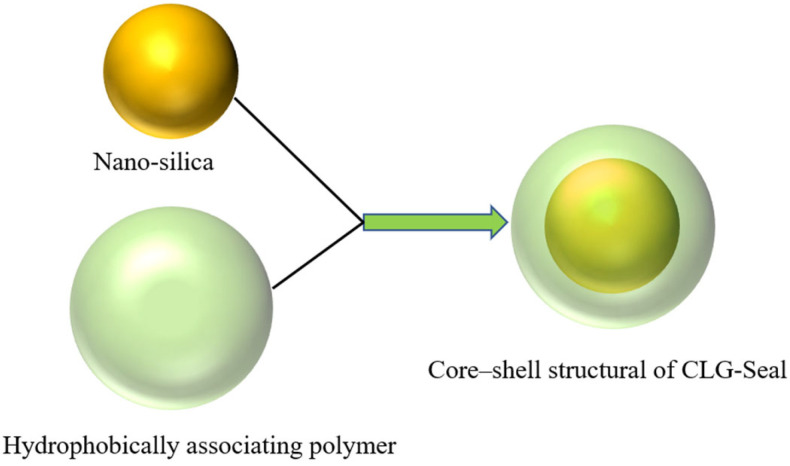
The microscopic molecular structural characteristic of CLG-Seal.

The molecular structure of the newly developed nano-micro sealant CLG-Seal exhibits distinct core–shell structural characteristics, as shown in Figure 11. The inorganic nano-silica constitutes the rigid core of CLG-Seal, which guarantees its plugging performance and effectively decreases the invasion depth of water-based drilling fluid. The hydrophobically associating polymer which is coated on the surface of nano-silica constructs the flexible shell of CLG-Seal, endowing the nano-micro sealant CLG-Seal with excellent deformability, adhesion film-forming capacity and perfect dispersibility.

When the nano-micro sealant CLG-Seal is applied to water-based drilling fluids, the polymer chains with hydrophobic association endow the nano-micron particles with favorable hydrophilicity and lipophilicity. After dissolving in aqueous medium, the polymer presents a large steric hindrance structure, which enables the CLG-SEA to exhibit excellent dispersion performance in water-based drilling fluids. The experimental results also reveal that the CLG-SEA possesses favorable dispersion properties in aqueous solution. When it is incorporated into water-based drilling fluids, CLG-SEA gains more opportunities to contact and enter into the nano-micron-scale pores of shale, thereby maximizing the plugging efficiency.

**Figure 12:**
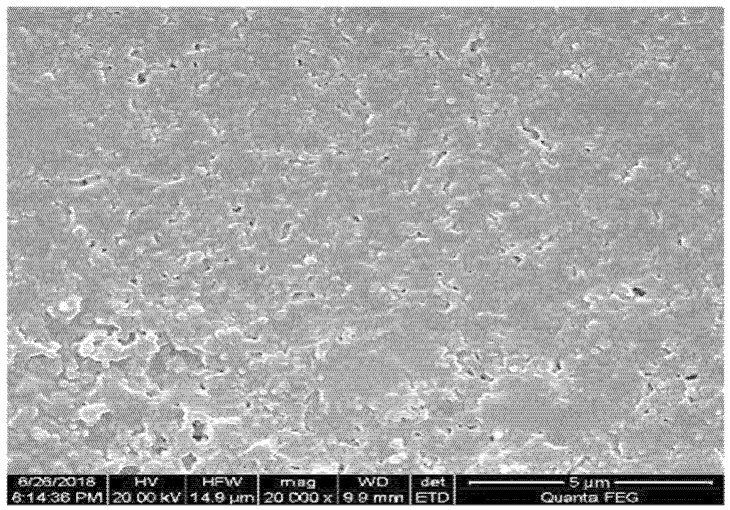
The SEM image of the gel film with a cross-linked network structure formed by CLG-SEA on the surface of shale rock.

Figure 12 presents the SEM image of the gel film with a cross-linked network structure formed by CLG-SEA on the surface of shale rock. When the CLG-Seal particles are close to each other, such as in the throat or filter cake of drilling fluid, the polymer chains with a flexible long chain structure that are coated on the surface of nano-silica constructs tend to be intertwined, forming a cross-linked network structure of gel film, thereby increasing the interaction between nano-micron particles and forming an impermeable plugging film.

In addition, due to the nanoscale effect, the CLG-Seal has a strong tendency to adsorb onto the surface of shale rock through hydrogen bonding with the shale matrix. The hydrophobically associating polymer which is coated on the surface of nano-silica with high elastic modulus and excellent mechanical properties can enhance the pressure-bearing capacity of the filter cake through elastic deformation. Therefore, these nano-micron particles can form a strong sealing film on the filter cake and at the micropores of shale rock, thereby creating a dense mud cake on the outside of the shale formation.

Finally, the nano-micro sealant CLG-SEA can enter the shale pores through the elastic deformation of the outermost layer of the structure, while the rigid nano-silica particles can prevent the filtrate of the drilling fluid from further entering the pores, forming a dense filter cake inside the nano-micron pores of the shale.

### 2.8. Field Application

Field application of the newly developed CLG-Seal was conducted in the third section (5060–5400 m) of Well WY3-2-3HF. Approximately 1.5% CLG-Seal was incorporated into the drilling fluid, and the fundamental property test results are summarized in Table 2.

The rheological properties (including apparent viscosity, plastic viscosity, and yield point) and gel strengths of the field drilling fluid remained relatively stable throughout the third section of Well WY3-2-3HF, as indicated in Table 2. The HTHP fluid loss was maintained below 2.4 mL, and the actual drilling fluid density ranged from 2.15 to 2.17 g/cm^3^.

**Figure 13:**
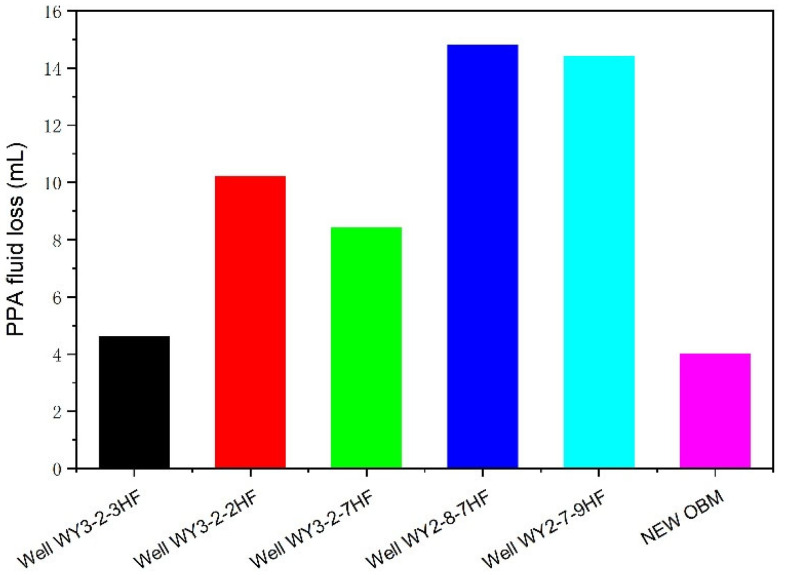
Evaluation of sand disk plugging performance for the field application interval.

Figure 13 presents a comparative analysis of PPA fluid loss between the field drilling fluid used in Well WY3-2-3HF and that of adjacent wells (WY3-2-2HF, WY3-2-7HF, WY2-8-7HF, WY2-7-9HF), as well as a laboratory-prepared oil-based drilling fluid.

As illustrated in Figure 13, the PPA fluid loss for the field drilling fluid in the third section of Well WY3-2-3HF was 4.6 mL. This represents a significant reduction compared to adjacent Wells WY3-2-2HF and WY3-2-7HF, decreasing by approximately 54.9% and 45.2%, respectively. Furthermore, the fluid loss is drastically lower than that recorded in early-stage Longmaxi Formation operations in this block (e.g., 14.8 mL for WY2-8-7HF and 14.4 mL for WY2-7-9HF). These results demonstrate that the drilling fluid employed in the third section of Well WY3-2-3HF exhibits exceptional plugging performance.

**Figure 14:**
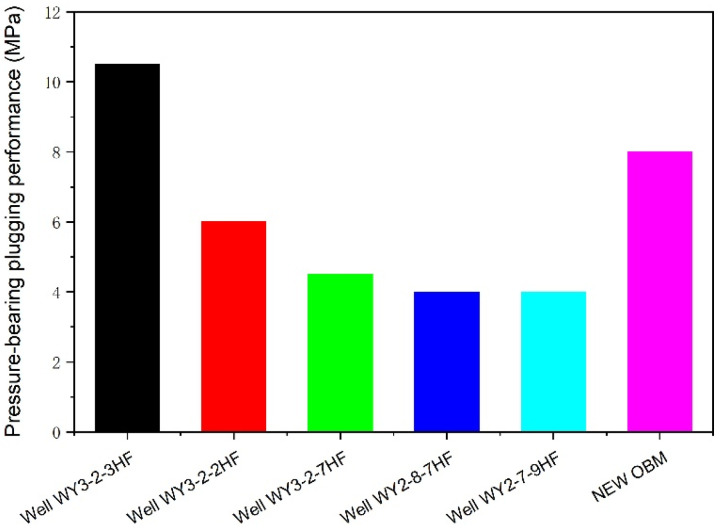
Evaluation of pressure-bearing plugging performance for the third section of Well WY3-2-3HF.

As demonstrated in Figure 14, the forward pressure-bearing capacity of the drilling fluid in the third section of Well WY3-2-3HF reached 10.5 MPa. This value represents a substantial improvement over adjacent wells and signifies a marked enhancement compared to the plugging performance achieved in the Longmaxi Formation of the Weirong Block during early-stage operations.

During the field trial, the third section of Well WY3-2-3HF was drilled to completion without any downhole complications, such as lost circulation or wellbore instability. The operation achieved a net drilling time of 42 h and an average rate of penetration (ROP) of 7.81 m/h. Notably, the mud weight was reduced by approximately 0.08–0.10 g/cm^3^ compared to adjacent wells. These results demonstrate that the newly developed CLG-Seal performed effectively under field conditions.

## 3. Conclusions

(1) An innovative high-temperature-resistant deformable nano-micron plugging agent, designated as CLG-Seal and featuring a “core-mantle-shell” structure, was successfully developed via laboratory synthesis. Comprehensive characterization using TEM and SEM confirmed that the particles exist predominantly at the nano-micron scale. Furthermore, particle size distribution (PSD) analysis revealed a relatively narrow distribution, with a median diameter (D_50_) of 146 nm and a D_90_ of 344 nm. These results demonstrate that the median particle size (D_50_) of CLG-Seal is tunable.
(2) Results from the low-permeability sand disk plugging test (PPA test) indicate that the fluid loss volume decreases progressively with increasing concentrations of the newly developed CLG-Seal after hot rolling at 150 °C. This demonstrates that CLG-Seal significantly mitigates fluid loss in low-permeability micro-fractures. Notably, a concentration of merely 3% is sufficient to achieve robust plugging performance.
(3) Results from both the displacement tests and the etched glass micromodel experiments demonstrate that the 4% CLG-Seal base fluid exhibits exceptional capability in plugging pores and fractures. This superior performance is primarily attributed to the unique “core-mantle-shell” structure of the agent. Specifically, the deformable outer shell provides adaptability to fissure walls, while the rigid nano-silica core (characterized by small particle size, high strength, excellent thermal stability, and non-hydration properties) ensures structural integrity under high pressure. Consequently, upon exposure to new formation, the agent rapidly plugs micro-pores and fractures matching its particle size, forming a thin yet dense filter cake. Furthermore, due to the high surface energy of the ultrafine particles, inter-particle adsorption and bridging effects facilitate the sealing of pores larger than the individual particles themselves. Additionally, the hydrophobic segments within the polymer matrix create a steric barrier that impedes the passage of polar water molecules through the non-polar film, thereby minimizing high-temperature, high-pressure (HTHP) fluid loss. This mechanism not only enhances the formation pressure-bearing capacity but also contributes significantly to reservoir protection and wellbore stability.
(4) Field trials of the newly developed CLG-Seal were conducted in the third section of Well WY3-2-3HF. The results indicate that the PPA fluid loss of the drilling fluid in this section was 4.6 mL, representing a significant reduction compared to adjacent wells and an even more substantial decrease relative to formulations used in the Longmaxi Formation of the same block during early-stage operations. Moreover, the forward pressure-bearing capacity of the drilling fluid reached 10.5 MPa, demonstrating a marked improvement over adjacent wells and confirming excellent plugging performance. During the field trial, the third section of Well WY3-2-3HF was drilled to completion without any downhole complications, such as lost circulation or wellbore instability. The operation achieved a net drilling time of 42 h and an average rate of penetration (ROP) of 7.81 m/h. Notably, the mud weight was reduced by approximately 0.08–0.10 g/cm^3^ compared to adjacent wells. These findings demonstrate that the newly developed CLG-Seal performed effectively under field conditions.

## 4. Materials and Methods

### 4.1. Synthesis Method and Procedure

Raw materials were weighed and pre-mixed in four separate beakers: Beaker 1 contained methyl methacrylate (70 g), acryloyl morpholine (20 g), deionized water (45 g), and sorbitan monooleate (2.3 g), which were homogenized to form Solution A; Beaker 2 contained 2-acrylamido-2-methylpropane sulfonic acid (6 g), acrylamide (2 g), sorbitan monooleate (1 g), alkyl phenol polyoxyethylene ether (0.5 g), and deionized water (30 g), which were homogenized to form Solution B; Beaker 3 contained deionized water (15 g) and azobisisobutyramidine hydrochloride (0.2744 g), which were dissolved to form Solution C; and Beaker 4 contained sodium bicarbonate (0.3 g), tetraethyl orthosilicate (4.2 g), hydrophilic nano-silica (1.2 g, AEROSIL 200 (Chengdu Kelong Chemical Co., Ltd., Chengdu, China), Germany, particle size: 12 nm), and deionized water (30 g), which were blended and subsequently subjected to ultrasonication for 20 min to form Solution D. Solution D was then charged into a 500 mL three-necked flask equipped with a stirrer and heated to 70 °C under a nitrogen atmosphere, followed by purging for 30 min. Subsequently, Solution A and 9 mL of Solution C were simultaneously added dropwise to the flask over 40–50 min, and the reaction mixture was held at 70 °C for 50–70 min. Following this, Solution B and the remaining portion of Solution C were added dropwise to the flask within 30 min. The temperature was then elevated to 80–85 °C and maintained for 4 h. Finally, the mixture was cooled to room temperature to afford the final product, designated as CLG-Seal.

All chemical reagents (methyl methacrylate(MMA), acryloyl morpholine(ACMO), sorbitan monooleate (Span 80), 2-acrylamido-2-methylpropane sulfonic acid(AMPS), acrylamide(AM), alkyl phenol polyoxyethylene ether (OP-10), azobisisobutyramidine hydrochloride (AIBA), sodium bicarbonate (NaHCO_3_), tetraethyl orthosilicate (TEOS), hydrophilic nano-silica) applied in the synthetic process were purchased from Chengdu Kelong Chemical Co., Ltd. and used as chemical purity grade or higher.

### 4.2. Evaluation of Sealing Performance in Low-Permeability Sand Disks

**Figure 15:**
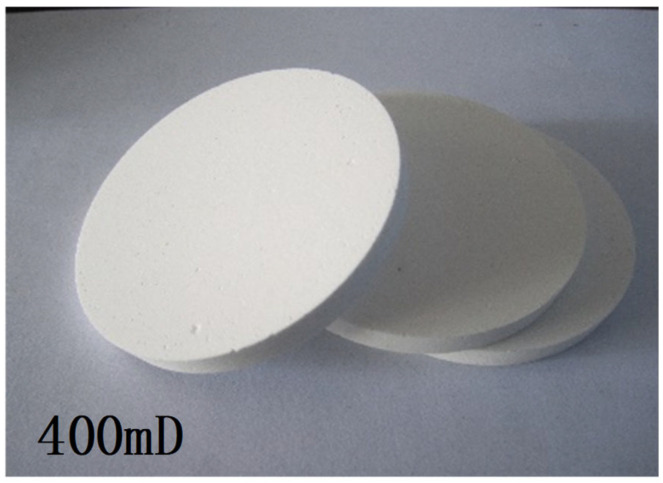
Schematic of the 400 mD low-permeability sand disk apparatus.

The sealing performance in low-permeability sand disks (PPA sand disks) was evaluated using a standard testing apparatus (Figure 15) under the following conditions: Filter disk No. 12114301d (400 mD permeability), 7 MPa pressure, and 150 °C aging for 16 h.

The base fluid formulation consisted of 4.0% bentonite slurry + 0.15% FA367 + 0.25% 80A51 + 4% SMP-3 + 3% SPNH + 4% SY-A01 + 1.5% SY-A07 + 0.3% polyamine, weighted to 2.1 g/cm^3^ with barite.

0#: Base fluid
1#: Base fluid + 1% CLG-Seal
2#: Base fluid + 2% CLG-Seal
3#: Base fluid + 3% CLG-Seal

### 4.3. Experiment on Displacement and Plugging Performance Evaluation

(1) Experimental Apparatus

**Figure 16:**
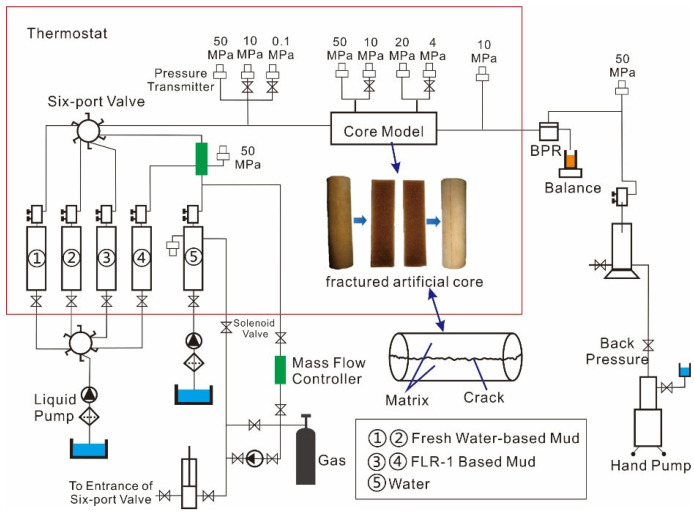
Schematic diagram of the plugging displacement experiment.

Displacement experiments were conducted on artificially fractured cores to simulate plugging behavior. The schematic of the experimental setup and its underlying principle are illustrated in Figure 16. The primary components of the system include a core holder, two high-precision plunger pumps, a thermostatic chamber, an intermediate pressure vessel, a gas flow meter, pressure transducers, a back-pressure valve, a vacuum pump, and the artificial fractured core.

(2) Experimental Procedure

① Artificially fractured cores (or natural cores) were weighed (*M*1) and subsequently evacuated for 12 h using a vacuum pump. Table 3 shows the experimental results of relevant parameters for artificial cores.

② Deionized water was introduced into the vacuum chamber, pressurized to 10 MPa, and maintained for 12 h to ensure complete saturation of the core sample.

③ The pressure was released, and the saturated core was retrieved. After wiping off surface moisture, the core was weighed again (*M*2). The porosity was then calculated according to Equation (1).

(1) $$\Phi =\left(M_{2}-M_{1}\right)/\rho V_{t}\times 100\%$$

where *Φ* represents the porosity, %; *M*_1_ and *M*_2_ denote the dry and wet weights of the core sample, respectively, g; *ρ* is the density of deionized water, g/cm^3^; and *V* denotes the bulk volume of the core sample, cm^3^.

④ The experimental core was placed in the core holder of the displacement apparatus, and a confining pressure of 3.5 MPa was applied prior to commencing the displacement experiments with the pre-configured fluids.

⑤ The digital acquisition system recorded the inlet and outlet pressures, and the differential pressure was utilized to evaluate the plugging efficiency of the fluid systems.

⑥ The resistance factor and residual resistance factor were calculated using Equations (2) and (3), respectively.

(2) $$R_{es}=P_{p}/P_{w}$$

(3) $$R_{res}=P_{s}/P_{w}$$

where *R_es_* and *R_res_* represent the resistance factor and residual resistance factor, respectively; and *P_p_*, *P_s_*, and *P_w_* denote the displacement/plugging pressure, subsequent water flooding pressure, and base fluid displacement pressure, respectively, with units of MPa.

**Table 3:** Experimental results of relevant parameters for artificial cores.

Core Parameters	Length/cm	Radius/cm	Initial Permeability/10^−3^ μm^2^	Permeability After Fracturing/10^−3^ μm^2^	Dry Weight/g	Wet Weight/g
Values	7.41	2.5	15	36	68.57	76.49

### 4.4. Evaluation of Plugging Performance in Etched Glass Micromodels

(1) Experimental Apparatus

**Figure 17:**
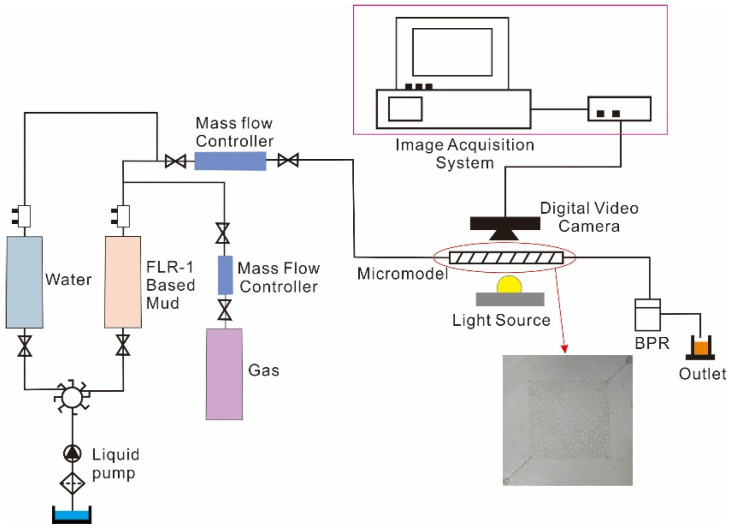
Schematic of the experimental apparatus for plugging evaluation in etched glass micromodels.

A plugging simulation experiment was conducted using an etched glass micromodel. Displacement tests were performed on a 4% CLG-SEAL aqueous solution to observe and analyze the plugging behavior of the newly developed nanomicron-scale plugging agent from a microscopic perspective. A schematic of the experimental setup is presented in Figure 17.

The primary components of the system comprise a displacement unit, a vacuum pump, an etched glass micromodel, and a video image acquisition system. The experimental setup utilized a standard glass micromodel with dimensions of 80 mm × 80 mm × 6 mm and an average pore-fracture width of 0.020 mm. A Nikon Model L110 digital camera (Nikon Corporation, Tokyo, Japan) was integrated into the microscope system to record the visualized plugging behavior during the displacement process.

(2) Experimental Procedure

① The experimental apparatus was assembled according to the schematic, and a back pressure of 1.5 MPa was applied.

② The etched glass micromodel was evacuated using a vacuum pump and subsequently saturated with water at an injection rate of 0.2 mL/min.

③ The prepared fluid was displaced into the micromodel under a controlled back pressure of 1.5 MPa.

④ The flow behavior within the micromodel was recorded using the video image acquisition system.

⑤ Upon completion of the displacement test, the micromodel was retrieved for post-experimental observation of the plugging status within the pores and fractures using a high-power microscope (Nanjing Kathmatic Technology Co., Ltd., Nanjing, China).

## Figures and Tables

**Table 1:** Calculated resistance factors and residual resistance factors.

Nano-Micro Sealant	Base Slurry Displacement Pressure/MPa	Plugging Pressure/MPa	Subsequent Water Flooding Pressure/MPa	Resistance Factor	Residual Resistance Factor
CLG-Seal	0.15	0.77	0.46	5.13	3.07
N-Seal	0.15	0.45	0.50	3.00	3.33
SD-Seal	0.15	0.31	0.29	2.07	1.93

**Table 2:** Fundamental properties of the field drilling fluid in the third section of Well WY3-2-3HF.

Well Depth/m	AV (mPa·s)	PV (mPa·s)	YP (Pa)	Gel Strength (10 s/10 min)/Pa	HTHP FL/K	Density (g/cm^3^)
5062	80	69	13	4/12	2.2/0.5	2.15
5196	81	68	13	5/11	2.4/0.5	2.16
5390	79.5	66	12	4/10	1.9/0.5	2.17
Design value	≤90	≤70	5–15	3–7/8–13	4/1	1.75–2.20

## Data Availability

The raw/processed data required to reproduce these findings cannot be shared at this time as the data also form part of an ongoing study.
